# A Novel Iterative MLEM Image Reconstruction Algorithm Based on Beltrami Filter: Application to ECT Images

**DOI:** 10.3390/tomography7030026

**Published:** 2021-07-28

**Authors:** Abdelwahhab Boudjelal, Abderrahim Elmoataz, Bilal Attallah, Zoubeida Messali

**Affiliations:** 1 Image Team, GREYC Laboratory, University of Caen Normandy, CEDEX, 14050 Caen, France; abderrahim.elmoataz@unicaen.fr; 2 Electrical Engineering Laboratory LGE, M’sila University, 28000 M’sila, Algeria; bilal.attallah@univ-msila.dz; 3 Electronics Department, University of Mohamed El Bachir El Ibrahimi—Bordj Bou Arréridj, 34030 Bordj Bou Arréridj (BBA), Algeria; messali.zoubida@gmail.com

**Keywords:** EM algorithm, beltrami filtering, emission-computed tomography, FBP algorithm, poisson distribution

## Abstract

The implementation of emission-computed tomography (ECT), including positron emission tomography and single-photon emission-computed tomography, has been an important research topic in recent years and is of significant and practical importance. However, the slow rate of convergence and the computational complexity have severely impeded the efficient implementation of iterative reconstruction. By combining the maximum-likelihood expectation maximization (MLEM) iteratively along with the Beltrami filter, this paper proposes a new approach to reformulate the MLEM algorithm. Beltrami filtering is applied to an image obtained using the MLEM algorithm for each iteration. The role of Beltrami filtering is to remove mainly out-of-focus slice blurs, which are artifacts present in most existing images. To improve the quality of an image reconstructed using MLEM, the Beltrami filter employs similar structures, which in turn reduce the number of errors in the reconstructed image. Numerical image reconstruction tomography experiments have demonstrated the performance capability of the proposed algorithm in terms of an increase in signal-to-noise ratio (SNR) and the recovery of fine details that can be hidden in the data. The SNR and visual inspections of the reconstructed images are significantly improved compared to those of a standard MLEM. We conclude that the proposed algorithm provides an edge-preserving image reconstruction and substantially suppress noise and edge artifacts.

## 1. Introduction

Image reconstruction is a type of inverse problem [1], and as the main and most complex issue related to such problems, the crucial information of an image is not consistently attainable. Analytical and iterative reconstruction algorithms are the two principle types of methods recommended in the literature [2,3,4]. Filtered backprojection (FBP) [5] is the most highly investigated diagnostic method used in emission-computed tomography (ECT) [6,7], and uses single photons, i.e., single-photon emission-computed tomography (SPECT) reconstruction [8], and pairs of photons, i.e., positron emission tomography (PET) [9] reconstruction. As a result of its rapid and simple application in software reconstruction programs, the supremacy of FBP has been upheld for a high number of applications. Analytic image reconstruction processes typically contain numerous restrictions, which damage and restrain their effectiveness. On the whole, its analytic methodology disregards measurement noise and commonly produces images that have unsatisfactory compromises in terms of spatial resolution and image modifications.

By using iterative image reconstruction algorithms, it is possible for these inadequacies to be avoided [10,11,12,13,14,15]. In comparison to analytic reconstruction methods, iterative reconstruction methods have significant benefits regarding their capability to integrate different solutions to image-degrading issues such as unfinished, noisy, and dynamic datasets in a more efficient manner. By incorporating the benefits of iterative reconstructions, significant results are achieved, enabling the completed outcome to have a higher standard of qualitative elements, as well as a more precise approximation of the tracer concentration, and enhanced and upgraded spatial resolution and image contrast.

Within ECT, the most commonly used iterative algorithms are the maximum-likelihood expectation maximization (MLEM) [16,17,18] algorithm, and the enhanced and quicker ordered subset EM (OSEM) algorithm [19]. However, it is important to acknowledge that iterative reconstruction methods individually are insufficient in reconstructing artifact-free images, and additional developments are therefore necessary to achieve enhanced outcomes.

Based on the above understanding of past works [20,21,22,23,24], this paper proposes a novel scheme based on iterative filtering for computing the MLEM algorithm for ECT image reconstruction from noisy projections, namely a filtered MLEM. More precisely, we include an additional Beltrami [25,26,27] filtering step at each iteration of the MLEM to reduce noise and unwanted artifacts while preserving the edge information. The combination of these techniques not only preserves the geometrical structures, such as edges, it also achieves the best reconstruction results and performance in terms of the resolution.

In Section 2 of this paper, we describe the main steps of the proposed image reconstruction method using Beltrami image filtering and provide a solution using the MLEM algorithm. Section 3 presents the evaluation criteria for the quality of the reconstruction. Numerical results from a quantitative comparison of recent reconstruction methods in terms of the relative norm error, SNR, and human visual quality of the reconstructed images are given in Section 4. Finally, Section 5 provides some concluding remarks regarding this work.

## 2. Materials and Methods

In the MLEM algorithm, the measured projection datasets play a significant role. In a SPECT scanner, the size of the projection data depends on both the number of detectors in the camera strip and the number of angles. If the camera contains *b* number of detectors and we measure at *a* angles, then the number of elements of projection data vector, J=a×b. For easier calculation, this vector is generally represented as a column vector. In PET, there is a ring of detectors around the patient that measures the annihilation event. If *N* is the number of detectors in the ring, then J=N(N−1)/2 N is the number of all pairs of the detector in coincidence. For a tomographic reconstruction, the image to be reconstructed is digitized into a matrix *x* with $n_{x}$ rows and $n_{y}$ columns. Again, for computational purposes, we represent the image as a column vector with *I* elements, where $I=n_{x}\times n_{y}$ elements. Physicists have proved that such emissions follow a Poisson model [28]. Therefore, the unknown total number of emission events in the *i*th pixel, $\hat{x}\left(i\right)$, represents a Poisson random variable, with mean $\bar{x}\left(i\right)$.

We know that a system matrix represents the probability distribution of the projection data. Hence, elements of system matrix p(i,j) represent the probability of emission *i* to be detected by detector *j*. It is possible to calculate the expected value of the projection data depending on the system matrix using the following formula [17,18,29]:

(1) $$y\left(j\right)=E\left[x(j)\right]=\sum_{i=1}^{I}x(i)\cdot p(i,j)$$

Because x(i) are independent Poisson random variables, a linear combination of these variables is also distributed as in the above Poisson equation. Regarding the above equation, the probability of the detected data is

(2) $$L\left(x\right)=P\left(y|x\right)=\prod_{j=1}^{J}\frac{e^{-\hat{x}\left(j\right)}\hat{x}{\left(j\right)}^{y(j)}}{y(j)!}$$

The likelihood function L(x) indicates the Poisson probability of observing the given counts in detector pairs in coincidence if the true density is x(i). The log-likelihood function is produced through the combination of Equations (1) and (2) [30]:

(3) $$\begin{array}{cc} l\left(x\right) & =log\left(L\left(x\right)\right)\\ & =\;\sum_{j=1}^{J}\sum_{i=1}^{I}x_{i}p_{i,j}+\sum_{j=1}^{J}y_{j}\log\left(\sum_{i=1}^{I}x_{i}p_{i,j}\right)\;-\;(\sum_{j=1}^{j}log(y_{j}!)) \end{array}$$

It can be seen using the first and second derivatives of the log-likelihood function that the matrix of second derivatives is negative semi-define, and that l(x) is concave [30]. As a result, the conditions sufficient for vector $\hat{x}$ to yield the maximum of *L* are the Kuhn-Tucker (TK) conditions [31]:

(4) $$\begin{array}{cc} 0= & x\left(i\right)\frac{\partial l(x)}{\partial x(i)}{|}_{\bar{x}}\;\\ & =\;-\hat{x}\left(i\right)p\left(i,\cdot \right)+\sum_{j=1}^{J}\frac{n\left(j\right)\hat{x}\left(i\right)p\left(i,j\right)}{\sum_{\overset{´}{i}=1}^{I}\hat{x}\left(\overset{´}{i}\right)p\left(\overset{´}{i},j\right)} \end{array}$$

and

(5) $$\frac{\partial l(x)}{\partial x(i)}{|}_{\bar{x}}\leq 0\ldots \;if\;\hat{x}\left(i\right)=0$$

The MLEM algorithm starts with an initial estimate $x^{\left(0\right)}$, and uses the maximization condition to iteratively improve the estimate. Researchers have used a variety of initial estimates to reach the results faster [32,33,34]. The main formula for the MLEM algorithm is derived by solving the above maximization condition for $\hat{x}\left(i\right)$, given in iteration *n* + 1:

(6) $$x^{n+1}\left(i\right)=x^{n}\left(i\right)\frac{1}{\sum_{i=1}^{I}p\left(i,j\right)}\sum_{j=1}^{J}\frac{n(j)p(i,j)}{\sum_{\overset{´}{i}=1}^{I}x^{n}\left(\overset{´}{i}\right)p\left(\overset{´}{i},j\right)}$$

Analyzing Equation (6), the MLEM algorithm can be summarized as follows:

1. Start with estimate $x^{\left(0\right)}$, where $x^{\left(0\right)}>0$ for i=1,2,3,…,I.
2. If $x^{\left(n\right)}$ denotes the estimate of *x* at the $n^{th}$ iteration, define a new estimate $n^{\left(n+1\right)}$ using Equation (6).
3. If the required accuracy for the numerical convergence has been achieved, then stop; else, return to (2).

### 2.1. Noise Reduction Method Based on Geometric Flow

To identify a suitable resolution for ECT image reconstruction, the iterative filtering structure of the MLEM method is used as a result of uniting an enhanced image-filtering methodology into the existing MLEM iteration strategy, specifically, an existing iterative filtering strategy that endorses and supports the MLEM image quality during the iterative development. As a result of upholding the key aspects, the vast proportion of dimensions functioning within the image are entrenched into an improved dimension that enables the use of the influential disparity geometry operatives [25].

An inventive geometric dispersal flow method is the Beltrami flow, which has the objective of lowering the image area manifold and motivating the flow in the direction of a minor surface resolution, while simultaneously upholding the edges.

The Beltrami framework, is based on a nonlinear flow that was applied as an edge-preserving denoising and deblurring algorithm for signals and especially multi-channel images.

The manner in which the Beltrami flow is communicated is shown below [35]:

(7) $$x_{t}=\frac{1}{\sqrt{g}}\mathrm{div}\left(\frac{\nabla x}{\sqrt{g}}\right)$$

where $x_{t}=\frac{\partial x}{\partial t}$ represents the derivative of the density of image *x* with consideration of time *t*. In addition, ∇x is the gradient vector, which is $\nabla x\equiv (x_{x_{1}},x_{x_{2}})$, for 2D images; however, $\nabla x\equiv (x_{x_{1}},x_{x_{2}},x_{x_{3}})$ for 3D volumes, where $x_{x_{1}}=\frac{\partial x}{\partial x_{1}}$ indicates the derivative of *x* with consideration of $x_{1}$ ($x_{2}$ and $x_{3}$ have similar circumstances). Moreover, div is the divergence operator, signified for vector function $f=(f_{x_{1}},f_{x_{2}})$ as

(8) $$\mathrm{div}\left(f\right)=\frac{\partial f_{x_{1}}}{\partial x_{1}}+\frac{\partial f_{x_{2}}}{\partial x_{2}}$$

Lastly, *g* is used to signify the determinant for the initial essential form of the surface, which is represented as $g=1+{\left|\nabla x\right|}^{2}$. The format of *g* originates from a prompted metric for the Euclidean (n+1)−D space within which the concentration of an n−D image is entrenched within the dimension [25] (with n=2 for 2D images, and n=3 for 3D volumes). The purpose of *g* is to deliver the measurement of the area increase amid surface domain *S* and image domain *x*, and consequently is a substantial element in encouraging the flow in the direction of the surface with the smallest surface area.

Furthermore, $1/\sqrt{g}$ in Equation (7) acts as an edge signifier. Consequently, the objective of the Beltrami flow is to function as a selective noise filtering strategy that upholds the edges and lowers the dispersal across the edges, while applying wide-ranging and substantial diffusion everywhere else [36]. As can be seen through Equation (7), the application of a partial differential equation is founded upon the limited dissimilarities, using a Euler forward dissimilarity estimation for *x*, and central dissimilarities to estimate the spatial offshoots:

(9) $$x^{k}=x^{k-1}+h_{t}\frac{x_{x_{1}x_{1}}\left(1+x_{x_{2}}^{2}\right)+x_{x_{2}x_{2}}\left(1+x_{x_{1}}^{2}\right)-2x_{x_{1}}x_{x_{2}}Ix_{x_{1}x_{2}}}{{\left(1+x_{x_{1}}^{2}+x_{x_{2}}^{2}\right)}^{2}}$$

where $x^{k}$ is the reconstructed image in the kth iteration, $h_{t}$ is the time step, and $x_{x_{1}}$ is the first derivative with respect to $x_{1}$ (applied in the same way as $x_{2}$), i.e.,

(10) $$x_{x_{1}}\left(i,j\right)=\frac{x(i,j+1)-x(i,j-1)}{2}$$

Here, $x_{x_{1}x_{1}}$ is the second-order derivative with respect to $x_{1}$ (applied in the same way as $x_{2}$), i.e.,

(11) $$x_{x_{1}x_{1}}\left(i,j\right)=x\left(i,j+1\right)-2x\left(i,j\right)+x\left(i,j-1\right)$$

and $x_{x_{1}x_{2}}$ is the mixed double partial derivative with respect to $x_{1}$ and $x_{2}$:

$$\begin{array}{c} x_{x_{1}x_{2}}\left(i,j\right)=\frac{x(i+1,j+1)-x(i+1,j-1)}{4}\\ \;-\frac{xI(i-1,j+1)+x(i-1,j-1)}{4} \end{array}$$

These derivatives are calculated from the reconstructed image in the past iteration $x^{k-1}$, where i,j are the pixel indices.

### 2.2. Combining MLEM Algorithm and Beltrami Image Flow Filtering

In this section, we propose a new scheme called filtered MLEM (*f*-MLEM). MLEM algorithm was combined with Beltrami image flow filtering to reduce the noise and unwanted artifacts with preserving the edge information which is the filtered MLEM algorithm for the unpenalized problem by including an additional filtering step in each iteration. As is well known, the MLEM algorithm is an iterative approach that increases the likelihood of the estimated image in each iteration. Our proposal is to introduce a filtering step for each iteration through which the current estimated image is smoothed in a suitable manner.

The MLEM method was applied sequentially by changing the number of iterations *K* of the Beltrami filter in each iteration *N* of the MLEM algorithm. We started the MLEM iterations with a large *K* to converge at the desired solution. With an increase in the number of iterations of the MLEM algorithm, we decrease the number of iterations of the Beltrami filter. These two types of iterations have an inverse relationship because, with an increased number of iterations of the MLEM algorithm, the image becomes more apparent, and we therefore no longer need further improvements and therefore reduce the number of iterations for the Beltrami filter. This leads to a reduction in memory usage and time consumption, while maintaining the image quality. This means that during the iterations, unknown parties are allowed to vary while the MLEM algorithm wish to maximize the likelihood function *L*, to obtain the estimated image $x_{i}$ the best fits the measured data $n_{j}$.

To obtain acceptable result images, owing to its edge-preserving performance, we applied a Beltrami filter within the pixel distance along the $x_{1}$ and $x_{2}$ directions:

$$h_{x_{1}}=0.5\cdot \left[\begin{array}{ccc} 0 & 0 & 0\\ -1 & 0 & 1\\ 0 & 0 & 0 \end{array}\right]$$

$$h_{x_{2}}=0.5\cdot \left[\begin{array}{ccc} 0 & -1 & 0\\ 0 & 0 & 0\\ 0 & 1 & 0 \end{array}\right]$$

$$h_{x_{1}x_{1}}=0.5\cdot \left[\begin{array}{ccc} 0 & 0 & 0\\ 1 & -2 & 1\\ 0 & 0 & 0 \end{array}\right]$$

$$h_{x_{2}x_{2}}=0.5\cdot \left[\begin{array}{ccc} 0 & 1 & 0\\ 0 & -2 & 0\\ 0 & 1 & 0 \end{array}\right]$$

$$h_{x_{1}x_{2}}=0.5\cdot \left[\begin{array}{ccc} 1 & 0 & -1\\ 0 & 0 & 0\\ -1 & 0 & 1 \end{array}\right]$$

The proposed *f*-MLEM reconstruction algorithm is summarized in Algorithm 1.
**Algorithm 1:** filtered MLEM Algorithm
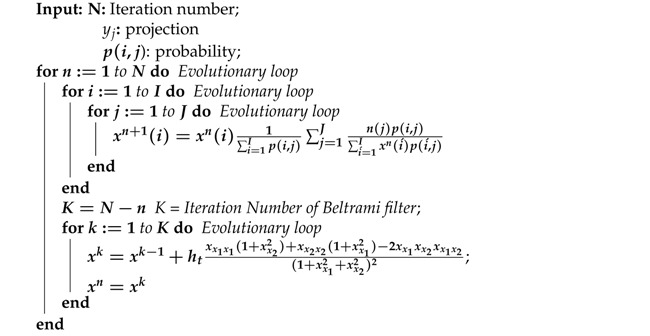


## 3. Performance Evaluation

To assess the overall performance of the proposed algorithm, computer simulations were conducted. To evaluate the reconstructed results objectively, two image-quality measurement parameters were computed: the relative norm error of the reconstructed images [36], which is defined as

(12) $$df=\frac{∥x-\hat{x}{∥}^{2}}{{∥x∥}^{2}}$$

The Signal-to-noise ratio defined as:

(13) $$SNR=\frac{\sum_{j=1}^{M}\sum_{i=1}^{N}{\left[\hat{x}\right]}^{2}}{\sum_{j=1}^{M}\sum_{i=1}^{N}{\left[x-\hat{x}\right]}^{2}}$$

The gray level value of the test image is denoted as *f*, with $\hat{f}$ being the same value as in the reconstructed image. A lower df value, and higher SNR values, indicate that the resulting reconstructed image is closer to the test image. The number of iterations is a further criterion contributing to the iterative reconstruction algorithm, with a smaller number of iterations being preferable. Comparisons of profiles lines displaying isolines of the reconstructed images and a plot of the profiles of reconstructed images are also used.

We simulated a PET scanner in a 2D mode, which has 420 crystals in each ring with each crystal having a cross-section of 6.3 × 6.3 mm${}^{2}$.

**Figure 1:**
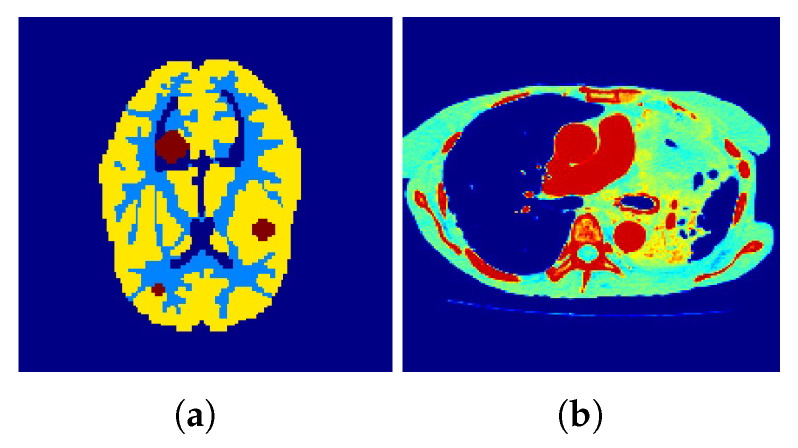
Input images: (**a**) Hoffman Brain Phantom and (**b**) standard medical image of an abdomen.

In our work, we use the phantoms shown in Figure 1. Figure 1a shows a Hoffman Brain Phantom [37], which was used to simulate the distribution of a PET tracer through gray matter, white matter, and three tumors. The digital designs of the tumors in the phantom provide essential information regarding the reconstructed images. The smallest tumor that can be individually distinguished indicates the resolution of the scanner. The second phantom, shown in Figure 1b, is a standard medical image of an abdomen [38], and provides an anatomically accurate simulation of the radioisotope distribution obtained in a healthy stomach. Quantitative and qualitative explorations of the influences of scatter attenuation that can be observed in imaging using SPECT, or alternatively, PET, can be enabled by the phantom. All the phantoms and additional reconstructed images have pixel resolutions of 192×192 pixels with a pixel size of 3 × 3 mm${}^{2}$. To create a sonogram, a simulated phantom image was forward projected and 30% true coincidences were uniformly introduced to simulate a background of random and scattered fractions. The fractions were determined by counting and dividing them by the total number. Poisson noise is usually generated separately to create noisy sinograms. Here, the noise added to the data measured 500 k events.

## 4. Results and Discussion

The objective of this section is to deliver a comprehensive comparison of the reconstruction outcomes of the regular MLEM algorithm with those of the proposed *f*-MLEM algorithm for the purpose of image reconstruction within emission tomography. To enable a clear view of the images, they were scaled to [01] and exhibited using an unchanged linear gray scale. Primarily, the foremost study is centered upon providing a comparison amid the noise greatness in low- and high-count areas of the MLEM and *f*-MLEM images. To ensure that the objective of the study was accomplished, the modification as a method of positioning for the phantom images reconstructed using the MLEM and *f*-MLEM algorithms was first carefully investigated. For the purpose of developing the projection information, Poisson noise was added to every attenuated phantom estimate. Following this, the noisy projections generated were used for the purpose of reconstructing the 2D phantoms.

**Figure 2:**
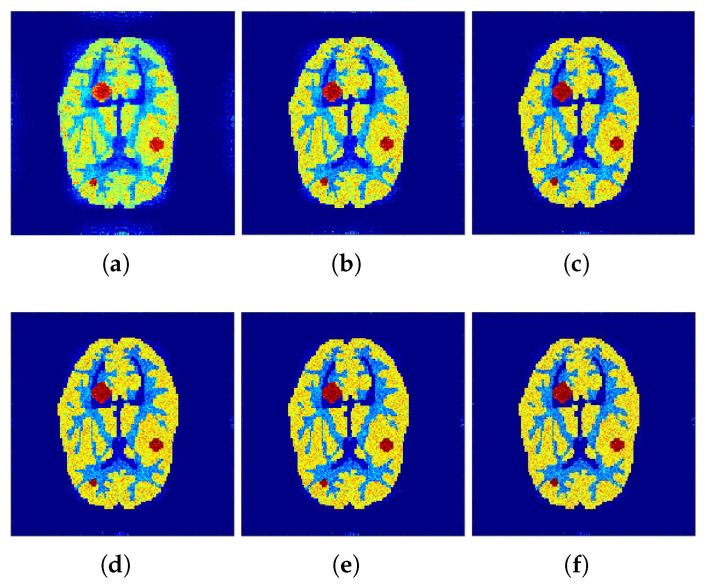
Conventional MLEM reconstructions. (**a**–**c**) row: iterations 20, 40, and 60. (**d**–**f**) row: iterations 80, 100, and 120.

**Figure 3:**
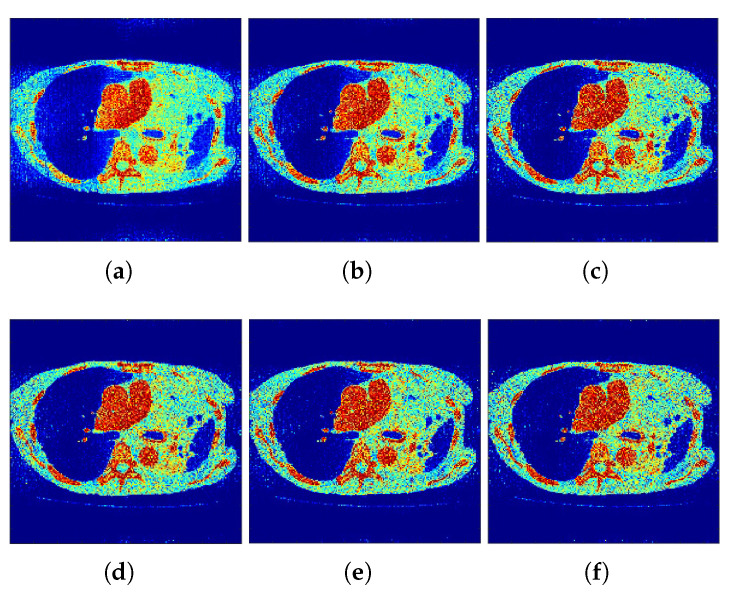
Conventional MLEM reconstructions. (**a**–**c**) row: iterations 20, 40, and 60. (**d**–**f**) row: iterations 80, 100, and 120.

Correspondingly, Figure 2 and Figure 3 show the reconstructed images that were generated and attained from the regular MLEM algorithm by altering the quantity of iterations for the Hoffman Brain and standard medical image of an abdomen phantoms. As the figures show, it is clear that the visual quality of the reconstructed images increased substantially by increasing the number of iterations.

**Figure 4:**
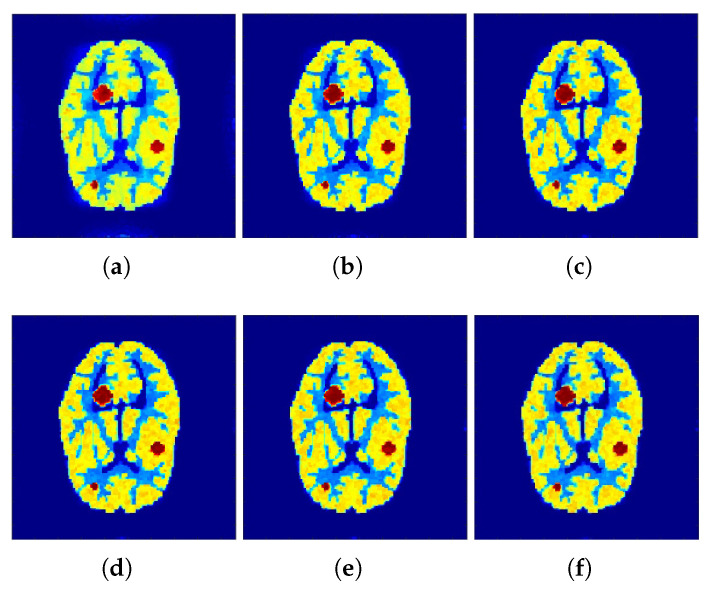
*f*-MLEM Intermediate MLEM reconstructions. (**a**–**c**) row: iterations 20, 40, and 60. (**d**–**f**) row: iterations 80, 100, and 120.

**Figure 5:**
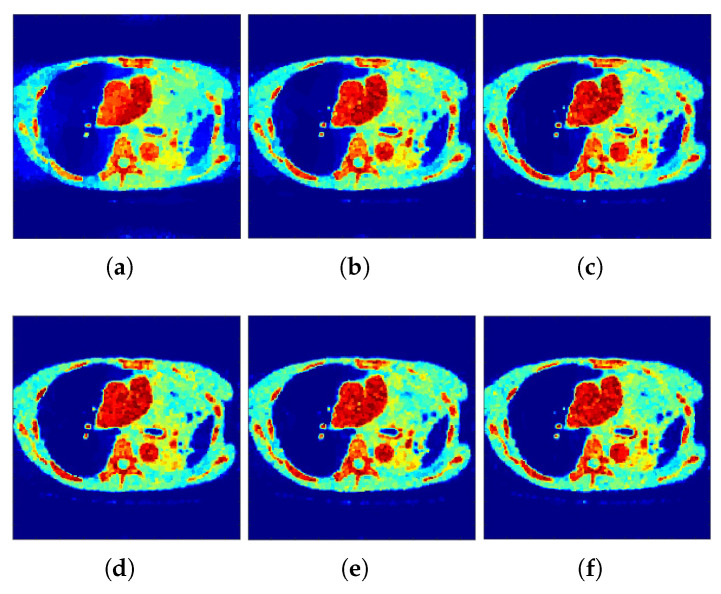
*f*-MLEM Intermediate MLEM reconstructions. (**a**–**c**) row: iterations 20, 40, and 60. (**d**–**f**) row: iterations 80, 100, and 120.

The reconstructed images that were generated and attained from the projected *f*-MLEM algorithm by altering the number of iterations are shown in Figure 4 and Figure 5. Based on these experiments, it is evident that the *f*-MLEM algorithm has the potential to successfully eradicate star artifacts produced by using the regular MLEM algorithm. In comparison to the MLEM algorithm, the excellence dimensions of *f*-MLEM are substantially improved. As a result of the evaluation between the two algorithms, it is possible to conclude that the projected algorithm is dependable and suitable for improving the overall standard of reconstructed images, and is a more appropriate strategy for regulating edge and noise artifacts. Figure 4a shows the reconstruction results of the *f*-MLEM algorithm at iteration 20. Figure 4b shows the results at iteration 40, at which our approximate feasibility criterion is fulfilled. An image with a higher contrast at iteration 60 is shown in Figure 4c. A considerable reduction in noise artifacts is shown for the iterative *f*-MLEM algorithm. Figure 4d shows the results at iteration 80, and Figure 4e shows the results at iteration 100 when applying a Beltrami filter at iteration 60. This latter method appears to achieve a better image in terms of the visual quality.

**Figure 6:**
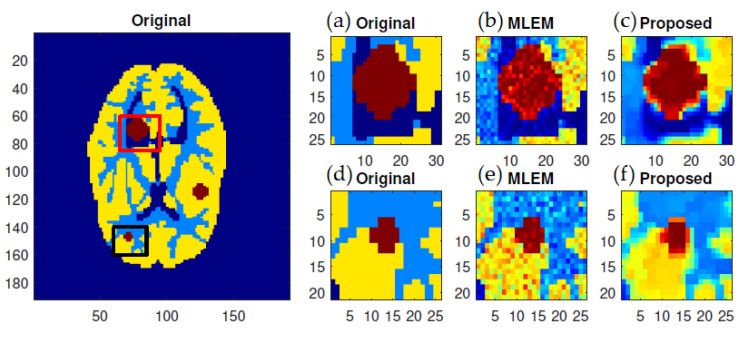
ROIs of Huffman Brain Phantom reconstruction achieved using various algorithms as shown under magnification: (**a**,**d**) the ground truth; (**b**,**e**) MLEM; and (**c**,**f**) *f*-MLEM.

**Figure 7:**
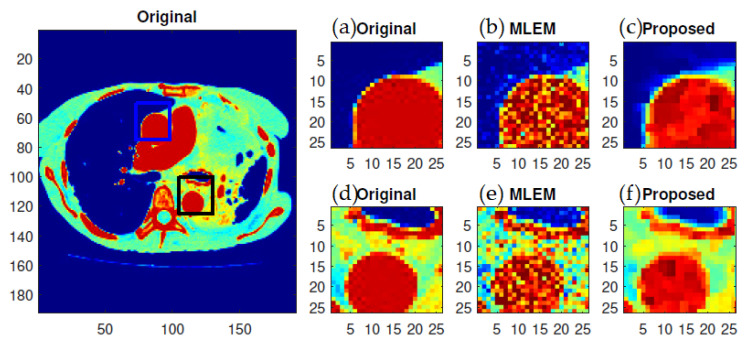
ROIs of standard medical image of abdomen reconstructed using various algorithms as shown under magnification: (**a**,**d**) the ground truth; (**b**,**e**) MLEM; and (**c**,**f**) *f*-MLEM.

The most noticeable difference when comparing the results from the MLEM and *f*-MLEM algorithms is that the *f*-MLEM reconstructed images are much smoother than the MLEM reconstructed images, and the former contain fewer artifacts. In particular, the artifacts are reduced in number within the peripheral regions of the reconstructed images. The reconstruction outcomes of the pair of simulated phantoms yielded the two rectangular areas illustrated in Figure 1a,b, which highlight how the two reconstruction algorithms differ. The proposed algorithm successfully preserved the edges. A detailed investigation reveals that the two image reconstruction algorithms differ. For example, the hot lesion edges were effectively preserved when applying the proposed algorithm, whereas artifacts and deviations were more likely to occur with the conventional MLEM algorithm. Furthermore, the intensity distribution within the hot areas was made more homogeneous by the proposed algorithm as compared to the conventional MLEM algorithm. Likewise, the proposed algorithm produced a better outcome for the Hoffman Brain Phantom. It was followed in efficiency by the conventional MLEM algorithm (Figure 6). A visual examination showed that the outcomes generated by the two algorithms for a standard medical image of an abdomen are not significantly different (Figure 7). It is clear from these findings that the proposed algorithm is more effective in generating smoother images with minimal bias and deviations compared to the conventional MLEM algorithm.

**Figure 8:**
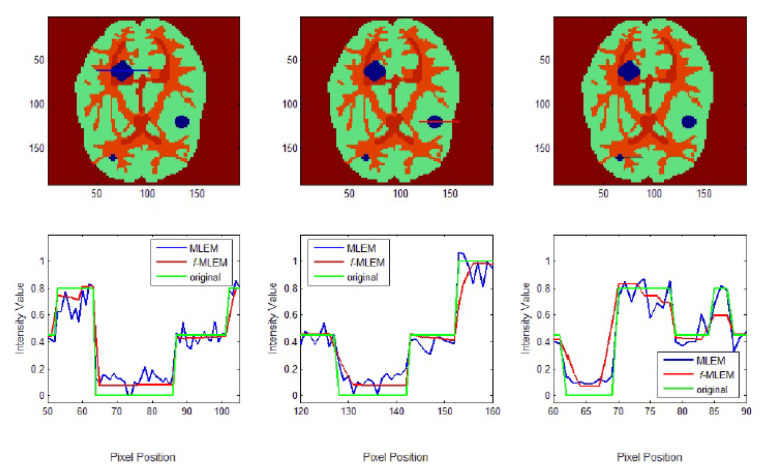
1D Line profile of two reconstruction algorithms across different RIOs.

To further display the differences, we used an image of a Huffman Brain Phantom and a standard medical image of an abdomen to examine the edge-preservation capability of the proposed algorithm. Horizontal 1D line profiles through the reconstructed images and the ideal Huffman Brain Phantom image, which includes three ROIs, are compared in Figure 8. These profiles were calculated using 80 iterations.

**Figure 9:**
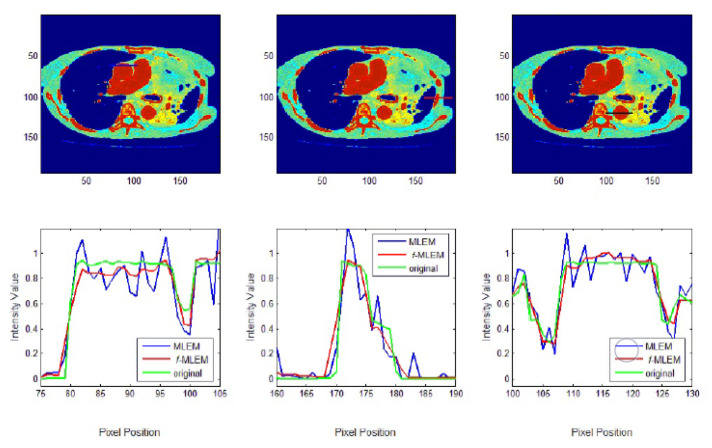
1D Line profile of two reconstruction algorithms across different RIOs.

The conventional MLEM algorithm allows spatial noise, and introduces some bias in the region with the same pixel values in the reconstructed image. The iterative MLEM algorithm produces an unbiased profile but a noisy image for the same number of iterations as used in the iterative *f*-MLEM. The standard medical image of the abdomen included three ROIs, and the line segments crossing the regions can be seen in Figure 9. From these profiles, we can see that the proposed algorithm lowers the noise in different regions while keeping the edges between regions sharp. It can be seen that there are nontrivial values when applying the conventional MLEM method for pixels outside the phantom, which is open air. With the proposed iterative *f*-MLEM algorithm, these aerial pixels are nullified, and their values are redistributed to the pixels inside the phantom, therefore causing their values to increase. Our newly proposed *f*-MLEM method outperforms the conventional MLEM in terms of reconstructing the ideal profile. Visually, the images provided by the proposed *f*-MLEM algorithm are close to the original image.

**Figure 10:**
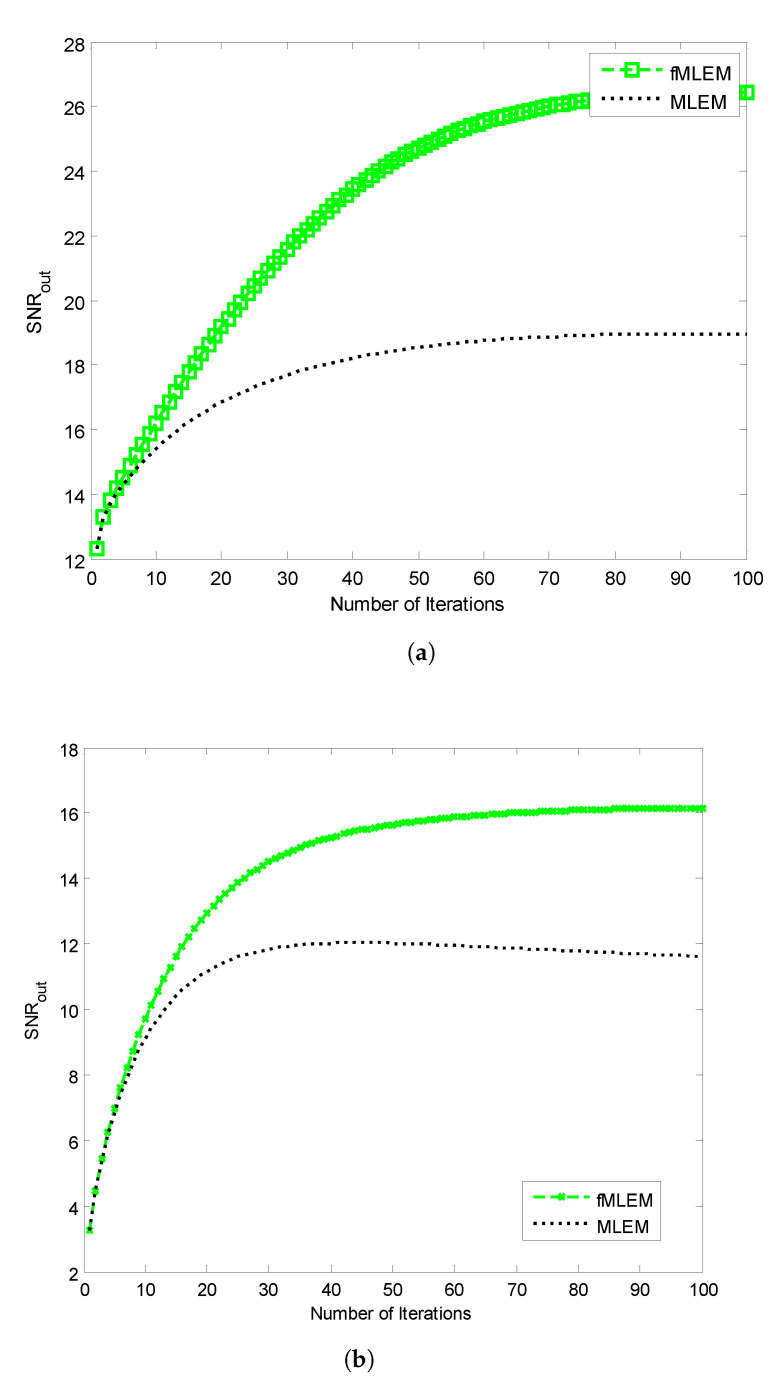
SNR vs. the number of iterations for (**a**) Huffman Brain and (**b**) Abdomen Phantom images.

The SNRs of the reconstructed images obtained using the conventional MLEM algorithm and the proposed *f*-MLEM algorithm versus the number of iterations are shown in Figure 10. The later demonstrates that the *f*-MLEM algorithm provides better quality measurements than the conventional MLEM algorithm. The iterations number is much required to enhance the image quality.

**Figure 11:**
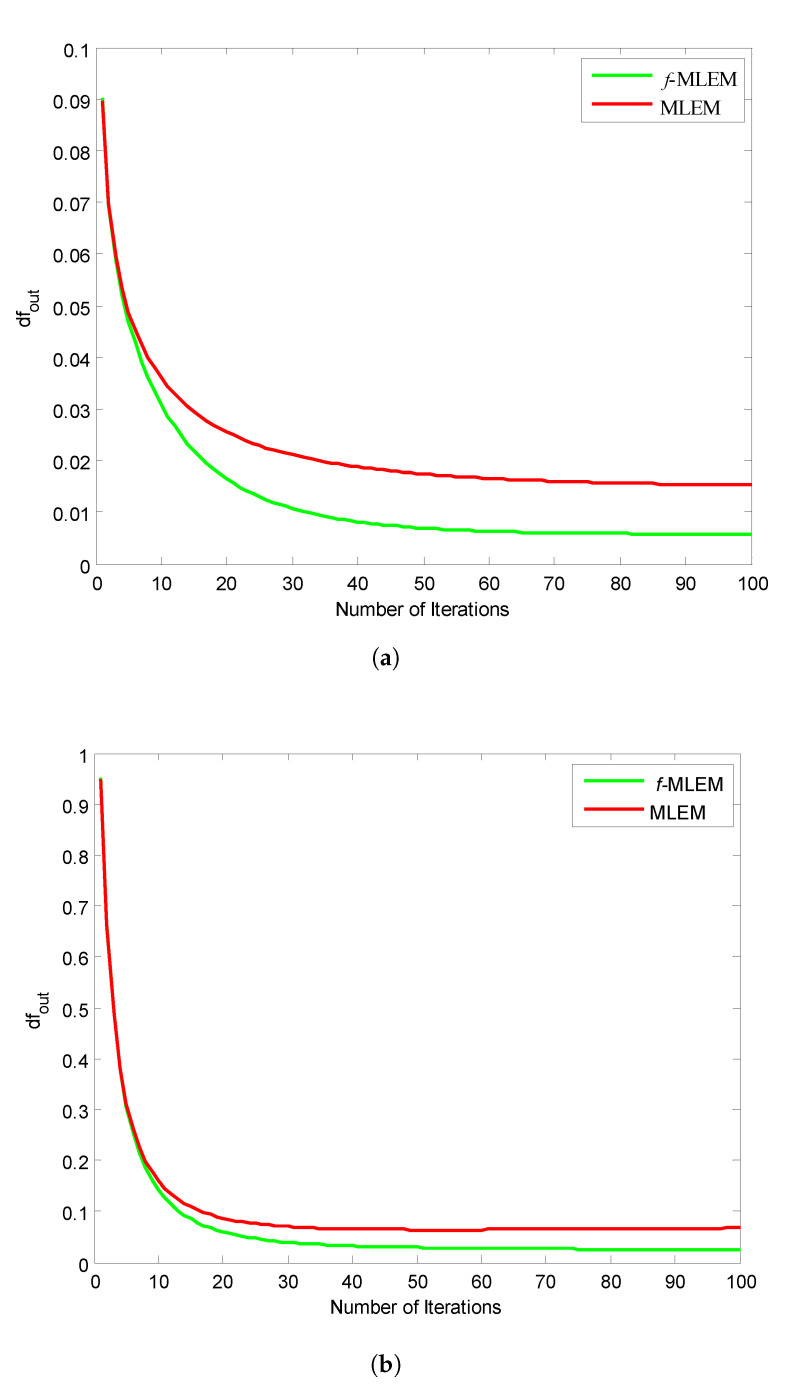
SNR vs. the number of iterations for (**a**) Huffman Brain and (**b**) Abdomen Phantom images.

Figure 11 shows the relative norm errors (df) versus the number-of-iteration curves for a Hoffman Brain Phantom image and a standard medical image of an abdomen when applying the proposed *f*-MLEM and the conventional MLEM algorithm. The proposed *f*-MLEM algorithm achieves better results even at a small number of iterations, and produces a better quality of reconstruction in terms of the relative norm errors.

It can easily be seen that the performance parameters are considerably improved compared to those of the conventional MLEM, particularly for 50 iterations, and the performance parameters remain almost constant after this number. It should be noted here that the number of iterations is necessary to improve and refine the quality of a reconstructed image. Both the quality measurements (SNR) and the relative norm errors (df) clearly reveal that the performance of the conventional MLEM algorithm after 100 iterations is similar to the performance of the *f*-MLEM at 22 iterations, as shown in Figure 10a and Figure 11a. The proposed algorithm requires a minimum of around 20 iterations to display an acceptable reconstructed image. This is the most common method for eliminating star artifacts that are usually generated with a conventional MLEM algorithm. The proposed *f*-MLEM algorithm is fast and efficient because it provides the best reconstructed images after a sufficiently small number of iterations.

To more illustrate the benefits of our algorithm, we provide Table 1 which compare the Signal-to-noise ratio SNR obtained via reconstruction techniques discussed in this work. Table 1 shows the difference between MLEM and *f*-MLEM algorithms of Huffman Brain Phantom in terms of performance parameters using different number of iterations. *f*-MLEM algorithm performs better even at limited number of iterations.

In all the visual-displays, the quality measurements and line plots demonstrate that the proposed *f*-MLEM algorithm outperforms the conventional MLEM algorithm. From the above observations, we can conclude that the proposed algorithm provides an edge-preserving image reconstruction and substantially suppress noise and edge artifacts present after a small number of iterations. It also extends the conventional MLEM algorithm in reconstructions from noisy random projections with a small number of iterations.

However, despite all these advantages, this new technology is still suffering from the drawback. One drawback of these proposed algorithms compared to conventional analytical solutions is their high computational complexity. However, due to the continuous improvement of computer technologies, effective programming methods and intelligent implementation techniques and other aspects of modeling, this deficiency has been partially surmounted.

## 5. Conclusions

For the reconstruction of projection data with inadequate iterations and noise, a filtered MLEM algorithm has been developed and applied. Under this specific scenario, the analytical capacity and test phantom reproduction outcomes of a Hoffman Brain Phantom illustrate that the proposed *f*-MLEM algorithm delivers a substantial enhancement in terms of reconstructed image standards and preciseness in comparison to a regular MLEM algorithm. Furthermore, the application and execution of the projected *f*-MLEM is extremely straightforward and avoids disrupting the physical models it defines. This study demonstrated that the proposed algorithm is an effective and successful method for enhancing the reconstruction standards and performance capabilities. Additionally, the data addition procedure used in the filtered MLEM algorithm has the capability to be added consistently to the OSEM algorithm.

## Figures and Tables

**Table 1:** Signal-to-noise ratio (SNR) by varying number of iterations for *f*-MLEM and MLEM algorithms. Test image: Huffman Brain.

	10	20	40	60	80	100
MLEM	15.00	16.50	18.00	18.20	18.45	19.00
*f*-MLEM	16	19.00	23.30	25.10	26.02	26.30
